# Immunometabolism: A Novel Therapeutic Target and Its Pharmacological Modulation for Intervertebral Disc Degeneration

**DOI:** 10.3390/ijms27031133

**Published:** 2026-01-23

**Authors:** Mengting Cheng, Yichen Liu, Moran Suo, Kaizhong Wang, Xin Chen, Zhonghai Li

**Affiliations:** 1 Department of Orthopedics, First Affiliated Hospital of Dalian Medical University, No. 5, Longbin Road, Dalian Development Zone, Dalian 116000, China; 2 The First Clinical College, Dalian Medical University, Dalian 116044, China

**Keywords:** intervertebral disc degeneration (IDD), immunometabolism, regenerative medicine, oxidative stress, mesenchymal stem cells (MSCs), biomaterials

## Abstract

Intervertebral disc degeneration (IDD) is a leading cause of low back pain (LBP) and imposes a substantial social and economic burden. Current treatments mainly relieve symptoms but rarely halt or reverse disc degeneration, and key gaps remain in our understanding of its pathophysiology. Accordingly, promoting intervertebral disc regeneration (IVDR) has been proposed as a potential therapeutic aim. Immunometabolism, which refers to the bidirectional interplay between immune responses and cellular metabolism, is increasingly recognized as a key factor affecting the balance of disc homeostasis and degeneration and has become an emerging research focus. In this review, we synthesize evidence supporting a dual and context-specific role of immunometabolism in IDD and IVDR. On the one hand, certain immune cells and anabolic cytokines or growth factors may promote a regenerative microenvironment by supporting disc cell survival and extracellular matrix (ECM) synthesis. On the other hand, pro-inflammatory mediators and metabolic disorders, including oxidative stress, mitochondrial dysfunction, and lipid or amino acid imbalance, drive a catabolic cascade that accelerates ECM breakdown and cellular senescence. We summarize current knowledge regarding key immune cell subsets, cytokine networks, and metabolic pathways implicated in IDD pathogenesis and IVDR, and we discuss how these immunometabolic principles are being leveraged in emerging interventions such as stem cell-based therapies, gene therapy, and advanced biomaterials. By integrating mechanistic insights with translational advances, this review aims to clarify actionable immunometabolic targets and to inform the rational development of regenerative strategies for disc-related diseases.

## 1. Introduction

Intervertebral disc degeneration (IDD) is a major contributor to chronic low back pain (LBP) and represents a common musculoskeletal condition [1]. The intervertebral disc is a fibrocartilaginous structure composed of the nucleus pulposus (NP), annular fibrosus (AF), and cartilaginous endplates (CEPs), connecting adjacent vertebral bodies [2,3]. The disc faces a progressive risk of loss of function due to a combination of aging, mechanical injury, and genetic factors. These factors cause harmful changes in the extracellular matrix (ECM), increased oxidative stress, and pro-inflammatory microenvironment [4,5,6], ultimately contributing to pain and functional impairment. Although current clinical strategies, such as rest, analgesic drugs, and surgical treatment, can alleviate the symptoms, they are generally unable to stop, delay, or reverse disc degeneration.

This viewpoint has been supported by converging evidence. For example, Silagi et al. stated that hypoxia-inducible factor (HIF) related signaling is important for maintaining the disc’s metabolic state of health [7]. In addition, Xiang et al. reported that exosomes from induced pluripotent stem cell-derived mesenchymal stem cells can mitigate disc degeneration partly through metabolic reprogramming of senescent nucleus pulposus cells [8]. These findings suggest that immunometabolic regulation can exert a major, but context-dependent, influence on disc fate. On the other hand, a homeostatic immunometabolic state, in which some immune cells and growth factors are active, promotes ECM synthesis and cell proliferation and restrains apoptosis [9]. Stabilized HIF signaling, together with balanced lipid and amino acid metabolism, helps maintain disc homeostasis [10]. On the other hand, an abnormal shift to a pro-inflammatory and catabolic state promotes the degeneration of the intervertebral disc. This process often includes immune cell infiltration and sustained release of proinflammatory cytokines, which activate matrix-degrading enzymes including matrix metalloproteinases (MMPs), accelerate ECM degradation, suppress matrix synthesis, and promote cell death [11].

This pathological state is due in part to disordered cell metabolism. In the context of IDD, this refers to the abnormal reprogramming of the metabolic pathways in disc resident cells and infiltrating immune cells. This dysfunction can be understood through the lens of immunometabolism, which describes the metabolic reprogramming that occurs during immune cell activation and function [12]. Different immune cell subsets possess distinct metabolic profiles that dictate their fate and function. For example, effector T cells rely on aerobic glycolysis for rapid proliferation and cytokine production, while memory T cells utilize mitochondrial oxidative metabolism for long-term survival. In IDD, metabolic imbalance is often characterized by excessive reactive oxygen species (ROS) generation, disrupted lipid handling, and altered amino acid utilization. These changes fail to support tissue homeostasis and can actively amplify inflammation and matrix catabolism, thereby creating a self-reinforcing degenerative cycle [13].

This complex relationship between immunity and metabolism has inspired new ideas for therapeutic intervention to reprogram the disc microenvironment and promote IVDR. Based on these specific mechanisms, new approaches for IDD have been explored, including stem cell-based therapies, gene therapy, and advanced biomaterials. Therefore, this review critically summarizes immunometabolic mechanisms that contribute to IDD progression and highlights how emerging interventions seek to modulate these pathways. In this way, this review aims to provide a clearer mechanistic basis and translational perspective for developing improved therapies for disc-related disease.

## 2. Intervertebral Disc Regeneration (IVDR)

The intervertebral disc is a specialized load-bearing structure. Its composition and microenvironment support function but also create substantial barriers to regeneration. The basic structural units include the central nucleus pulposus (NP), the outer concentric annulus fibrosus (AF), and the superior and inferior cartilage endplates (CEPs), as basic anatomical studies have shown [3]. The NP retains its gelatinous and hydrated state due to a high concentration of large and aggregating proteoglycans such as aggrecan, while the AF obtains tensile strength from a highly ordered and cross-linked network of collagen fibrils. Type I collagen is the predominant collagen in the outer AF, and the amount of type II collagen increases from the outer layer to the inner layer [3,8,14]. This composite architecture is able to bear complex biomechanical load, dissipate energy, and allow for spinal movement [15]. However, these same features constrain endogenous repair. As the body’s largest avascular tissue, the disc depends on nutrients and oxygen delivery by CEPs, which results in the constitutive hypoxic, acidic, and nutrient-poor microenvironment inside the NP [16,17]. Disc ECM, including collagens, proteoglycans, and other matrix proteins, is maintained in a constant state of synthesis and degradation. As a result, intervertebral discs exhibit low cellularity, poor nutrient supply, and oxygen deprivation [16]. This inherent characteristic is helpful for disc physiology but restricts self-repair and self-restoration. The resident nucleus pulposus and annulus fibrosus cell proliferative potential is also inherently restricted. All of these factors contribute to the difficulty of reversing IDD through endogenous repair alone [18].

At the molecular level, the disc ECM contains multiple collagen types. In addition to type I and type II collagens, types III, V, VI, IX, and XI also contribute to the fibrillar network [19]. Proteoglycans are also essential. Aggregated and multifunctional proteoglycans carry chondroitin sulfate and keratan sulfate chains, which bind water molecules to generate osmotic pressure and resist compressive load [20]. The ECM also contains adhesive glycoproteins, such as fibronectin and laminin, and elastic components such as elastin [14].

The path of IVDR is set by the pathogenic factors and processes that drive the degeneration of the intervertebral disc. Epidemiological studies summarized by Urban and Fairbank identify modifiable risk factors such as obesity, smoking, and excessive mechanical loading, which can increase disc stress and accelerate breakdown. These findings provide a rationale for lifestyle and biomechanical interventions alongside regenerative strategies [21]. At the center of what IVDR needs to combat is a transition from tissue anabolism to catabolism. This is because matrix dismantling enzymes are activated, mainly including matrix metalloproteinase (MMPs), and a disintegrin and metalloproteinase with thrombospondin motifs (ADAMTSs) [22]. Illien-Jünger et al. have shown that the main cause of this catabolic state is the pro-inflammatory microenvironment, as cytokines such as IL-1β and TNF powerfully induce disc cells to produce these enzymes and to promote irreversible degradation of collagens and proteoglycans [23].

IVDR, on the other hand, aims to reverse the imbalance between catabolism and anabolism through the induction of functional ECM synthesis. Longo et al. have synthesized evidence indicating that growth factors such as bone morphogenetic proteins (BMPs), growth differentiation factor 5 (GDF-5), transforming growth factor beta (TGF-β), and insulin-like growth factor 1 (IGF-1) can induce disc cells to increase the production of proteoglycans and type II collagen, both in vitro and in preclinical models [24]. For instance, Masuda et al. provided experimental proof that recombinant osteogenic protein 1 (rhOP-1), which belongs to the BMP family, significantly enhanced proteoglycan and collagen production in rabbit disc cells [25]. Therefore, targeted delivery or endogenous induction of anabolic signals is not only a means to reduce degeneration but also a basic biological approach to enable IVDR.

## 3. Immunometabolism

Immunometabolism means that the metabolic reprogramming process occurs during immune cell activation and functional differentiation [12]. Different immune cell subgroups have different metabolic characteristics, which directly influence their differentiation, activation, and function. For example, effector T cells mainly rely on aerobic glycolysis to provide the energy required for rapid proliferation and cytokine secretion, while memory T cells depend on mitochondrial oxidative metabolism and lipid oxidation for long-term survival and surveillance [26].

The main paths of immunometabolism are glycolysis, the TCA cycle, oxidative phosphorylation, the pentose phosphate pathway, fatty acid metabolism, and amino acid metabolism [27]. Metabolic pathways not only provide energy and biosynthetic precursors but also participate in signal transduction and epigenetic regulation by generating intermediates. For example, TCA cycle intermediate succinate can stabilize HIF-1α to increase pro-inflammatory gene expression [26].

Metabolic reprogramming is linked to immune cell function. A specific metabolic state can determine whether an immune cell is activated and polarized, and an immune cell that has been activated will in turn reshape its own metabolic state. This bidirectional regulation is at the core of m of immunometabolism [7,28,29,30]. In addition, such metabolic reprogramming is not limited to immune cells. It also includes resident cells within the intervertebral disc. When disc degeneration occurs, disc cells undergo obvious metabolic changes that are closely related to local inflammatory response and function decline [8].

**Figure 1:**
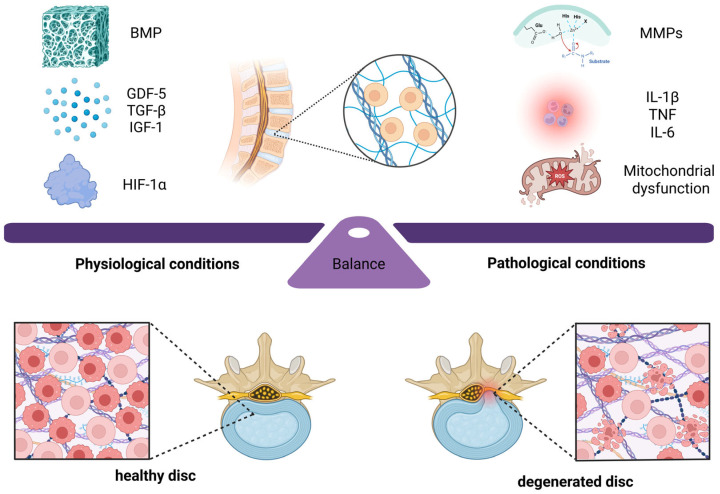
The immunometabolic regulation of intervertebral disc homeostasis and degeneration. The condition of the intervertebral disc is under the control of a dynamic and destructive balance of processes. Anabolic pathways are triggered by growth factors such as BMP, GDF-5, TGF-β, and IGF-1, together with HIF-1α, inducing the production of extracellular matrix (ECM) and tissue repair, thereby maintaining disc homeostasis and supporting intervertebral disc regeneration (IVDR). Catabolic processes are stimulated by pro-inflammatory cytokines (IL-1β, TNF, IL-6), and mitochondrial damage further results in increased expression of degradative enzymes (e.g., MMPs) and other matrix-degrading proteases (e.g., ADAMTS). This causes the ECM to degrade and induces chronic inflammation, which results in IDD.

When disc homeostasis is disrupted, a proinflammatory and catabolic environment is created. This disrupts the disc’s original immunometabolic condition and contributes to the initiation and persistence of IDD [31,32]. The dynamic balance between anabolic and catabolic immunometabolic signals is disturbed in IDD. On the one hand, anabolic signals such as GDF-5 and TGF-β can promote matrix production, alleviate inflammation, and promote disc regeneration [33]. On the other hand, proinflammatory cytokines such as IL-1β and TNF can inhibit matrix synthesis and accelerate the degeneration process [34] (Figure 1). 

## 4. Role of Immunometabolism in IDD

The intervertebral disc is maintained by a dynamic balance between anabolic (matrix-building) and catabolic (matrix-degrading) programs. Anabolic pathways, activated by growth factors such as BMPs, GDF-5, TGF-β, IGF-1, and HIF-1α, promote ECM synthesis and tissue repair, thereby supporting disc homeostasis and IVDR. In contrast, catabolic programs are driven by pro-inflammatory cytokines (e.g., IL-1β, TNF, and IL-6) and metabolic stressors such as mitochondrial damage, which increase the expression and activity of matrix-degrading enzymes (notably MMPs and ADAMTSs). This shift accelerates ECM breakdown and sustains chronic inflammation, ultimately promoting IDD. In degenerating discs, oxidative stress [35], the accumulation of detrimental lipid metabolites [6,36], and nutrient deprivation caused by impaired diffusion [13,17] further amplify catabolic signaling. These stressors directly activate key inflammatory signaling pathways such as NF-κB and MAPK, thereby upregulating pro-inflammatory cytokines (e.g., IL-1β and TNF) and catabolic enzymes (MMPs and ADAMTSs) [34,37]. Concurrently, inflammatory mediators reshape disc-cell metabolism. For example, pro-inflammatory signals can shift disc cells toward aerobic glycolysis, increase reactive oxygen species (ROS) production, and disrupt lipid and amino acid metabolism [8,13,28]. Importantly, this inflammation-driven metabolic reprogramming tends to favor the generation of biosynthetic and signaling intermediates that fuel inflammation, rather than supporting efficient matrix synthesis and repair. Together, this bidirectional crosstalk establishes a chronic, low-grade inflammatory microenvironment that continuously promotes ECM degradation, suppresses reparative processes, and accelerates cellular senescence [38,39]. This constitutes a core mechanism of IDD progression.

Overall, IDD pathogenesis is promoted by a vicious cycle between immune inflammation and metabolic dysregulation [7,28,29,30,38]. Within the degenerative microenvironment, infiltrating immune cells and elevated levels of pro-inflammatory mediators enhance ECM degradation through NF-κB and MAPK signaling and by upregulating matrix metalloproteinases. In parallel, they inhibit the synthesis of proteoglycans and type II collagen [34]. Metabolic abnormalities in disc cells not only impair cellular function but also further intensify the inflammatory response. For instance, lipid metabolism dysregulation can promote the release of harmful adipokines and is associated with cartilage mineralization and cell apoptosis [36]. This immunometabolic network therefore represents both a mechanistic hub of IDD and a set of actionable targets for intervention.

### 4.1. Role of Immune Cells and Cytokines in IDD

The association between chronic inflammation and IDD progression is strongly supported by human histopathological and molecular studies. Shamji et al. systematically analyzed cytokine profiles in surgically obtained human disc tissues. They found that herniated and degenerative discs exhibited significantly elevated levels of key pro-inflammatory cytokines, including IL-1α, IL-1β, IL-6, and TNF, compared with control discs that were not associated with pain [40]. Consistent with immune activation within the disc, Nakazawa et al. reported immune cell infiltration in degenerative human discs and identified distinct macrophage phenotypes, including M1, M2a, and M2c, within disc tissue [41]. This immune infiltration contributes to a local inflammatory milieu. Miyamoto et al. reported elevated mediators such as TNF, interleukins, MMPs, and prostaglandins within herniated discs, and these changes were associated with pain and reduced proteoglycan content [42]. These findings were later supported by Li et al. [43]. Collectively, these data support the rationale for developing IDD therapies by targeting specific components of the dysregulated immune response.

Marked differences in catabolic enzyme expression are observed between healthy and degenerative discs. In normal discs, MMP1 and ADAMTS4 are expressed at relatively low levels, whereas MMP3 and MMP13 are usually undetectable. In contrast, degenerated discs show much higher expression of MMP1, 3, 13, and ADAMTS4, and these increases tend to intensify with degeneration severity. This pattern supports their key role in disc matrix breakdown [44]. The production of these catabolic enzymes is largely induced by inflammatory mediators. IL-1β is a major driver in this context, and subsequent work has detailed IL-1-centered gene expression programs that promote matrix breakdown [37,45]. Mechanistically, IL-1β stimulates the production of MMPs and ADAMTSs while suppressing the synthesis of proteoglycans and type II collagen, thereby disrupting disc structure and exacerbating degeneration. During degeneration, local IL-1 levels increase and reshape both degradative enzyme expression and ECM composition [46]. Both IL-1α and IL-1β can activate NF-κB and MAPK signaling, increasing MMP and ADAMTS activity and accelerating ECM degradation [34,37,46]. TNF similarly functions as a catabolic cytokine and acts largely through NF-κB and MAPK signaling to induce MMP upregulation and disc cell death [34,37,46]. IL-6 can further amplify this inflammatory cascade, sustaining MMP expression and helping preserve a pro-catabolic microenvironment [19,42,47].

Other cytokines also contribute to ECM remodeling and phenotypic shifts. For example, Yao et al. showed in vitro that IL-17 treatment of human nucleus pulposus cells induced a dose-dependent increase in MMP-13 expression, reduced type II collagen, and increased type I collagen [39]. These changes collectively promote a fibrotic shift and disrupt the balance between ECM synthesis and catabolism.

Aging further compounds disc inflammation by promoting cellular senescence and accumulation of the senescence-associated secretory phenotype (SASP), which is difficult to clear in the avascular disc. SASP contains inflammatory cytokines and chemokines, including IL-1β, IL-6, and TNF, which collectively create and maintain a local inflammatory microenvironment. This milieu promotes senescence of neighboring disc cells and facilitates immune cell recruitment, reinforcing a vicious cycle of inflammation and degeneration [19,47]. Consistent with this mechanism, Rannou et al. showed that IL-1β stimulation of annulus fibrosus cells increased matrix metalloproteinase mRNA levels, enhanced PGE2 production, and promoted ECM breakdown in vitro [48]. Prostaglandins, especially PGE2, are inflammatory mediators implicated in radicular pain and can also accelerate matrix degradation, for example, by increasing MMP-3 activity, thereby destabilizing disc structure [49]. In addition, IL-8 has been reported to be elevated in degenerated discs. Degenerated discs also show reduced glycosaminoglycans and collagen content, suggesting a relationship between IL-8-related inflammation and IDD progression [50,51]. These observations support inflammation-focused strategies for treating disc-related pathology.

Macrophage plasticity is a pivotal determinant of disc inflammatory tone and tissue remodeling. Nakazawa et al. used immunofluorescence to demonstrate that macrophage subtypes, including M1, M2a, and M2c, accumulate within degenerated human disc tissue and increase with higher degeneration grades. This implies dynamic and stage-dependent immune involvement [41]. Using a coculture system, Yang et al. further demonstrated a bidirectional degenerative loop. Conditioned media from disc cells induced macrophages to upregulate inflammatory, catabolic, and chemokine-related genes, while conditioned media from M1 macrophages further increased catabolic and inflammatory gene expression in disc cells [52]. Macrophages can transition from an initial proinflammatory state, often described as M1-like, toward phenotypes associated with inflammation resolution and tissue repair, often described as M2-like, during acute injury responses [11]. However, chronic disc degeneration appears to maintain mixed and maladaptive macrophage signaling that perpetuates inflammation and matrix catabolism [41,52].

In summary, pro-inflammatory cytokines such as IL-1β, TNF, and IL-6, despite differing upstream triggers, largely converge on NF-κB and MAPK signaling. This convergence upregulates MMPs and ADAMTSs and suppresses collagen and proteoglycan synthesis, forming a central catabolic network that drives disc ECM degradation. These mediators do not act in isolation. Synergistic interactions and cascade effects exist, and IL-1β can induce TNF expression. However, the dominant cytokines at different stages of degeneration remain incompletely resolved, and much of the evidence still derives from in vitro and animal studies. Future work should define spatiotemporal cytokine programs in human discs and evaluate their potential as stratified therapeutic targets (Table 1).

### 4.2. Role of Metabolic Pathways in IDD

Metabolic dysfunction is a common hallmark of IDD and directly intersects with inflammatory signaling. Mitochondrial dysfunction can drive excessive ROS production, thereby causing oxidative stress that activates inflammatory responses and suppresses protective autophagy in disc cells [35]. Meanwhile, limited nutrient supply, due to the avascular nature of the disc and its dependence on diffusion through the cartilage endplates, forces nucleus pulposus cells to rely heavily on glycolysis. This promotes lactate accumulation and an acidic microenvironment [17]. Accordingly, disc cells reside chronically in hypoxic and acidic conditions, and under degenerative stress, glycolytic dependence and redox imbalance become more pronounced [17,54,55,56]. When ROS production exceeds antioxidant capacity, oxidative stress accelerates ECM degradation and apoptosis through inflammation activation and autophagy inhibition [35]. Furthermore, lipid metabolism dysregulation, including impaired fatty acid β oxidation and steroid hormone imbalance, can amplify oxidative stress and inflammatory signaling. This creates another self-reinforcing loop that promotes IDD progression. Disturbances in amino acid metabolism and calcium homeostasis can additionally impair ECM synthesis and disc cell viability, further aggravating degeneration [13].

Redox homeostasis is essential for normal cellular physiology [57], and mitochondrial dysfunction is a major source of pathological ROS accumulation. Key features of mitochondrial dysfunction include reduced mitochondrial mass, respiratory chain defects, opening of the mitochondrial permeability transition pore (MPTP), and loss of mitochondrial membrane potential (ΔΨm). Each of these can increase ROS generation. In disc cells, dysfunctional mitochondria therefore amplify oxidative stress and contribute to IDD progression [35]. When ROS generation exceeds ROS clearance by antioxidants, redox homeostasis collapses, and this is referred to as oxidative stress [58]. Excess ROS can damage macromolecules such as lipids, proteins, and nucleic acids, disrupt metabolic processes, and compromise cellular integrity [59].

Disc nutrient metabolism is also crucial for maintaining homeostasis and repair capacity. Degenerative changes in the cartilage endplates or annulus fibrosus can markedly reduce nutrient diffusion, increasing metabolic stress on nucleus pulposus cells. Under such conditions, strategies that reduce metabolic burden or improve metabolic efficiency may help limit nucleus pulposus apoptosis and slow IDD progression [60]. Because oxygen reaches the disc primarily by diffusion through the cartilage endplates, the disc center is particularly hypoxic. Anaerobic metabolism increases lactate accumulation and lowers pH [11,17]. Acidic conditions can impair cellular metabolism and are associated with increased ECM degradation and increased disc cell death, thereby worsening degeneration [13].

Beyond glycolysis and mitochondrial metabolism, disruption of the pentose phosphate pathway (PPP) can further reduce disc cell resilience under degenerative stress. The PPP is a major source of NADPH, which supports glutathione regeneration and antioxidant defense [57,58]. When PPP flux is reduced, antioxidant protection weakens. This makes degenerative disc cells more vulnerable to oxidative stress and to damage of proteins, lipids, and DNA [56,59]. This metabolic vulnerability suggests that maintaining PPP activity may represent an intervention node for improving metabolic resilience in IDD.

Lipid metabolism comprises digestion, absorption, synthesis, and breakdown of lipids, generating metabolites such as adipokines, fatty acids, and cholesterol [6]. Accumulating evidence indicates that lipid metabolism dysregulation contributes to IDD by promoting local inflammation, catabolic gene programs, and apoptosis [6]. More broadly, systemic dyslipidemia and altered lipid mediators can influence tissue inflammation and metabolic homeostasis [61]. Adipokines such as leptin appear to have context-dependent effects. They may exert proinflammatory actions that worsen degeneration. They may also show anabolic effects depending on microenvironmental cues [36]. Mechanistically, aberrant lipid handling, such as disordered fatty acid β oxidation, can lead to the accumulation of toxic lipid metabolites. This can trigger inflammation, endoplasmic reticulum (ER) stress, and oxidative stress, while also suppressing protective autophagy. Together, these changes drive ECM degradation, cell death, and even cartilage calcification [6]. Systemic hormonal shifts can further modulate these pathways. For example, postmenopausal estrogen decline has been linked to increased oxidative stress and ER stress in disc cells, increased ECM degradation and apoptosis, and reduced type II collagen. This may help explain the increased risk of IDD in postmenopausal women [62]. Therefore, correcting lipid metabolism dysregulation is a plausible therapeutic direction, although more detailed molecular mapping is still needed to enable precise targeting [6].

Finally, dysregulation of amino acid metabolism can disturb the balance between protein synthesis and breakdown, thereby impairing ECM maintenance and reducing disc cell viability. In a rabbit degeneration model, Sobajima et al. reported altered gene expression patterns, including reduced osteopontin-1 expression in degenerative discs [63]. Furthermore, elevated calcium levels in cartilaginous endplates are associated with reduced production and storage of type I and type II collagens and proteoglycans, as well as reduced endplate permeability. These changes further restrict nutrient transport and promote disc degeneration [13] (Table 2).

## 5. Role of Immunometabolism in IVDR

IVDR aims to restore disc structure and function by guiding cell survival, phenotype, and ECM production within a defined microenvironment. Achieving durable regeneration therefore requires coordinated control of anabolic signaling, inflammatory tone, and cellular metabolism. Growth factors can mitigate IDD by promoting disc cell proliferation, limiting apoptosis, enhancing ECM synthesis, and reducing catabolic inflammatory responses. These combined effects help reestablish matrix homeostasis and support disc repair. For example, members of the TGF-β family help maintain annulus fibrosus cell phenotype and can reduce postoperative disc cell death and matrix degradation [64,65]. Recombinant human osteogenic protein 1 (rhOP-1) has been shown to increase proteoglycan and type II collagen synthesis and to support matrix repair [25]. Because the disc is physiologically hypoxic, hypoxia-responsive regulators such as HIF-1α also contribute to IVDR by supporting disc cell survival and metabolic adaptation. In parallel, balanced lipid and amino acid metabolism provides both energy and essential building blocks for matrix biosynthesis, thereby directly influencing ECM homeostasis. For instance, HIF-1α can protect nucleus pulposus cells by promoting glycolysis and mitochondrial quality control, and adequate amino acid metabolism supplies substrates required for proteoglycan production [60]. Together, these observations highlight that regenerative cues and metabolic reprogramming can act synergistically. This framework supports the development of targeted IVDR strategies that combine pro-anabolic signaling with approaches that restore metabolic homeostasis and limit inflammation.

### 5.1. Role of Immune Cells and Cytokines in IVDR

The disc repair process is regulated by a network of growth factors and cytokines that promote cell proliferation, suppress apoptosis, and maintain ECM balance while attenuating inflammatory signaling. Among these mediators, TGF-β helps preserve annulus fibrosus cell phenotype, stimulates ECM production, and inhibits inflammatory reactions and matrix degradation. Consistent with these protective actions, TGF-β has been reported to support disc repair after discectomy [23,64,65]. Mechanistically, TGF-β signaling intersects with MAPK and NF-κB pathways within a complex regulatory network, which provides molecular targets for biologic therapies in IDD [66]. rhOP-1 significantly enhances proteoglycan and type II collagen production, in part by increasing cell proliferation in nucleus pulposus and cartilage endplate cells [25]. Other growth factors, including IGF-1 and basic fibroblast growth factor (bFGF), also contribute to disc repair by suppressing apoptosis and promoting proliferation of chondrocyte-like cells [67,68,69].

Disc cell metabolism and matrix anabolism are also shaped by local signaling that can act in autocrine and paracrine manners. For example, BMP7-mediated anabolism can be enhanced by transcriptional regulators such as FoxC2, indicating that intracellular regulatory programs can amplify growth factor-driven repair [9]. In tissue engineering settings, TGF-β1 can maintain annulus fibrosus cell morphology within scaffolds, promote cell proliferation and ECM synthesis, and reduce matrix degradation, cell loss, and inflammatory responses. TGF-β1 also participates in disc remodeling, and reduced TGF-β1 signaling has been associated with disc aging and degeneration [64,65]. Although discectomy is commonly used to treat symptomatic disc herniation, it can exacerbate degeneration by increasing resident disc cell death, damaging matrix structure, and increasing nitric oxide production. Supplementation with TGF-β3 has been reported to prevent or attenuate these adverse changes [23].

In an alginate bead culture model, Masuda et al. showed that rhOP-1 increased proliferation of rabbit nucleus pulposus and annulus fibrosus cells and enhanced the synthesis of proteoglycans and type II collagen [25]. In related work, Takegami et al. reported that rhOP-1 promoted matrix repair after chondroitinase ABC induced matrix damage, supporting its potential in disc regeneration [70].

Several growth factors have been shown to counteract apoptosis and reduce proteoglycan degradation. Gruber et al. demonstrated that IGF-1 exerts antiapoptotic effects in human intervertebral disc cells in vitro [69]. Nagano et al. reported that degenerated rat discs contained bFGF-expressing round chondrocyte-like cells with increased proliferative potential, suggesting that bFGF participates in the disc response to degeneration [67]. In addition, Liu et al. showed that urolithin A inhibited TNFα induced catabolic effects in nucleus pulposus cells and alleviated disc degeneration in vivo [68]. Other factors, such as epidermal growth factor (EGF), bFGF, and TGF-β3, have also been reported to promote ECM synthesis in vitro and in vivo, thereby limiting nucleus pulposus degeneration. In a large animal organ culture model, Illien Jünger et al. showed that growth factor treatment during surgery reduced nitric oxide levels, decreased disc cell death, and attenuated ECM degradation [23]. Cho et al. further reported that TGF-β can inhibit disc inflammation and protect ECM from degradation through MAPK and NF-κB signaling [66].

### 5.2. Role of Metabolic Pathways in IVDR

Metabolic pathways in disc cells support homeostasis and regeneration by enabling adaptation to hypoxia, maintaining redox balance, and sustaining lipid and amino acid metabolism. Together, these programs help preserve normal disc physiology and provide the biosynthetic capacity required for ECM repair. The intervertebral disc is largely avascular, and oxygen reaches the disc primarily by diffusion through the cartilaginous endplates from vertebral capillaries [13,71,72]. As a result, the nucleus pulposus and inner annulus fibrosus exist under low oxygen tension [13,73]. HIFs are principal regulators of cellular hypoxic responses. They function as heterodimers composed of oxygen-sensitive α subunits (α1, α2, and α3) and a constitutively expressed β subunit (HIF-β). Under hypoxic conditions, prolyl hydroxylase domain-containing enzymes are inhibited, which limits HIF-1α degradation and allows HIF-mediated transcriptional programs to proceed [13]. The effects of HIF-related signaling in disc biology can be context-dependent. For example, alterations in the PHD, HIF-1, and CA12 axis have been linked to changes in ECM synthesis in experimental disc degeneration models [74]. At the same time, HIF-1α has been shown to exert important protective functions beyond hypoxic adaptation. Madhu et al. reported that under hypoxia, HIF-1α promotes BNIP3 transcription and nuclear localization, thereby inducing mitophagy in nucleus pulposus cells. This process helps remove dysfunctional mitochondria and maintain metabolic homeostasis [75]. In subsequent work, genetic inhibition of HIF-1α or its downstream target BNIP3 in nucleus pulposus cells reduced ATP levels and lowered the NAD+/NADH ratio, further supporting the role of this axis in maintaining glycolytic energy production in these cells [76]. Complementary studies in other cell types have shown that HIF-1α overexpression can protect cells from stress induced death and promote anabolic functions [77,78,79,80]. These findings suggest that carefully enhancing HIF-1α signaling may help protect disc cells and support ECM synthesis, although the optimal degree and timing of activation remain to be defined for IVDR.

Balanced lipid metabolism provides necessary substrates for ECM synthesis and repair. Restoring physiological lipid homeostasis may therefore support disc regeneration by supplying energy and biosynthetic precursors for matrix production. Adiponectin and other adipokines have been reported to exert anti-inflammatory and pro-anabolic effects that can counteract a catabolic microenvironment [81]. Essential fatty acids contribute to cell membrane integrity and can act as signaling molecules that regulate repair [82,83]. Cholesterol also supports tissue regeneration by serving as a precursor for steroid hormones and as a component of cellular membranes [84]. Therefore, therapeutic strategies that correct lipid dysregulation may promote IVDR. Amino acid metabolism is similarly important for disc regeneration because it supplies substrates for protein synthesis and for the core proteins of proteoglycans. In particular, amino acids such as threonine, valine, and isoleucine contribute to rebuilding the integrity of the nucleus pulposus matrix [60,84,85,86,87].

### 5.3. Immunometabolic Targets, Translational Potential, and Challenges

The preceding sections highlight immune and metabolic mechanisms that can either promote or restrain IDD, and this mechanistic knowledge nominates several targets for IVDR. Because these targets sit at the intersection of inflammation and metabolism, they offer opportunities to interrupt the core vicious cycle that sustains degeneration [38].

One direct strategy is to inhibit pro-inflammatory cytokines to reduce catabolic signaling and matrix metalloproteinase activation, including neutralizing key drivers such as IL-1β and TNF [34,37]. However, achieving sufficient and durable local inhibition within the disc while minimizing systemic exposure remains challenging. Emerging delivery approaches, including targeted nanotherapeutics, may improve intradiscal retention and reduce off-target effects [88]. Conversely, enhancing anabolic growth factor activity, such as TGF-β, GDF-5, and BMP-7, can promote ECM synthesis more directly [24,25]. A major barrier is delivery control, since protein or gene delivery must be spatially and temporally controlled to avoid off-target effects, including ectopic bone formation or osteophyte development [87,89]. Reprogramming metabolic dysfunction represents another intervention layer. Potential approaches include HIF-1α stabilizers, antioxidants, and modulators of lipid metabolism, which may address cellular dysfunction and oxidative stress at a more fundamental level [13,35,75]. These strategies require high selectivity and should restore homeostasis without disrupting essential metabolic processes in resident disc cells. Modulating immune cell phenotype may also help shift the disc microenvironment toward repair. For example, redirecting macrophages from an M1-like proinflammatory state toward an M2-like reparative state could reduce chronic inflammation and support regeneration [11,41]. However, immune cell plasticity and heterogeneity within the disc microenvironment make such reprogramming difficult.

Several overarching translational hurdles also apply. The avascular, hypoxic, and acidic disc microenvironment limits drug bioavailability and reduces the survival and function of delivered cells [16,17]. In addition, current animal models often fail to reproduce the decades-long, multifactorial progression of human IDD, which complicates efficacy prediction [18,90]. Finally, patient stratification based on genetic background, immunometabolic signatures, and degeneration stage is likely to be essential for patient-specific and effective therapies [21,91]. Progress will therefore depend not only on selecting the right targets but also on developing advanced delivery systems, higher-fidelity models, and rigorous translational study designs.

## 6. Application of Immunometabolic Therapies in IVDR

IDD progression is promoted by disruption of the local immune microenvironment and dysregulation of disc-cell metabolism. In recent years, therapeutic strategies that focus on immunometabolic regulation have emerged in IDD research, including stem cell transplantation, gene (transgenic) technology, and biomaterial-based approaches. Collectively, these strategies aim to restore ECM homeostasis by modulating disc-cell energy metabolism (e.g., tuning glycolysis and limiting oxidative stress), suppressing pro-inflammatory mediators (e.g., IL-1β and TNF), and promoting anabolic synthesis (e.g., proteoglycans and type II collagen). However, to date, no single intervention that “only” targets energy metabolism has consistently produced a definitive and durable restoration of homeostasis; thus, reversing IDD is more likely to require multidimensional and synergistic therapies.

### 6.1. Application of Stem Cell Transplantation in IVDR

In recent years, stem cell transplantation has attracted considerable interest for IVDR [92,93]. Stem cell therapy may help restore intervertebral disc function and maintain disc structural stability [92,93]. Animal studies have also shown that stem cell therapy can promote IVDR, restore intervertebral disc height, and stimulate ECM formation, thereby improving biomechanical function [94,95,96,97,98]. Among candidate cell types, mesenchymal stem cells (MSCs) have been extensively investigated in vitro and in vivo for disc tissue repair [4]. Their therapeutic mechanisms are generally attributed to two major components: (i) differentiation potential and (ii) paracrine/immunomodulatory effects. For example, Steck et al. showed that human bone marrow-derived MSCs can be induced (with TGF-β) toward an intervertebral disc-like phenotype expressing nucleus pulposus-associated molecules such as type II collagen and aggrecan [99]. In addition, Miyamoto et al. reported that human synovial membrane–derived MSCs, in a co-culture system, reduced inflammatory factors (IL-1β, TNF) and key catabolic enzymes (MMP-3, ADAMTS-4) in rabbit nucleus pulposus cells, supporting an immunomodulatory/anti-catabolic role [100]. These observations align with the view that MSC-based therapy for IDD relies on combined regenerative and immunomodulating properties [4].

Conservative management with drugs and physiotherapy is usually adopted as the first choice for early-stage IDD [101,102]. If conservative treatment fails, surgery may be considered. However, conventional approaches largely focus on symptom control (pain relief) and mechanical stabilization and typically do not halt or reverse the underlying degenerative process [103]. Accordingly, stem cell transplantation and bioengineering technologies have become major research focuses and are considered promising options for disc degeneration [104]. Stem cells have shown substantial potential for repairing and regenerating damaged discs, supported by many in vitro studies, animal experiments, and early clinical investigations [92,93,94,95,96,97,98,100,105,106,107,108,109,110].

An increasing number of studies have progressed toward early-phase clinical translation, and most current strategies focus on MSCs, sometimes combined with biological scaffolds [104]. Nevertheless, during disc degeneration, upregulated cytokines and endogenous proteases create a hostile microenvironment for transplanted cells [13,16,55]. In particular, excessive MMP activity and/or an imbalance between MMPs and their inhibitors (TIMPs) can accelerate ECM breakdown and impair tissue remodeling [111]. Therefore, boosting TIMPs and restoring the catabolic–anabolic balance has been proposed as a potential supportive direction for IVDR [112].

Beyond cells themselves, MSC-derived small extracellular vesicles (sEVs)/exosomes have been reported to downregulate catabolic markers (e.g., ADAMTS-4, MMP-3) and upregulate anabolic markers (e.g., aggrecan and collagen), suggesting a cell-free therapeutic avenue [113]. Clinically, Yoshikawa et al. reported that autologous bone marrow–derived MSC transplantation may promote disc regeneration in degenerative disc disease [105]. Watanabe et al. found that MSCs increased the proliferative capacity of human nucleus pulposus cells in co-culture [108]. Meisel et al. reported that cell-based interventions were associated with reduced low back pain, maintenance of disc height, and increased water content in adjacent discs [106,109]. Coric et al. further supported the feasibility of cell-based disc repair within a clinical trial framework [107]. Overall, these early studies suggest acceptable safety profiles and potential symptom/structure benefits, but definitive efficacy remains to be established.

Stem cell transplantation can enhance proteoglycan synthesis and thus support IVDR [99,114,115,116]. Korecki et al. reported increased proteoglycan production in co-cultures of chondrocytes and MSCs [114]. Steck et al. found that MSCs induced with TGF-β produced type II collagen and proteoglycans resembling nucleus pulposus–like cells [99]. Chen et al. showed that co-culturing synovial MSCs with annulus fibrosus cells under increasing concentrations of TGF-β promoted differentiation toward a nucleus pulposus–like phenotype and increased proteoglycan synthesis [115]. Le Visage et al. observed increased proliferation in both nucleus pulposus and annulus fibrosus cells when co-cultured with MSCs, while enhanced proteoglycan production was more evident in annulus fibrosus cells [116]. These in vitro co-culture findings provide a mechanistic rationale for intradiscal cell injection as a repair strategy.

Induced pluripotent stem cells (iPSCs) can be differentiated into nucleus pulposus-like cells, offering another potential route for disc regeneration [117]. Extracellular vesicles released by MSCs (MSC-derived exosomes) act as intercellular messengers and have been explored for disc repair [118]. Exosomes have been reported to ameliorate IDD through antioxidant and anti-inflammatory effects and by reducing endoplasmic reticulum stress in nucleus pulposus cells [119]. For instance, exosomal miR-223 has been reported to reduce LPS-associated detrimental responses while upregulating ECM-related genes (aggrecan, type II collagen) and downregulating matrix-degrading enzymes (ADAMTS-4, MMP-3, MMP-13) and NF-κB–related proteins [4].

A major translational obstacle for stem cell therapy in IDD is identifying an optimal cell source. Autologous disc-derived stem cells are hard to get: getting stem cells from healthy discs is iatrogenic and constrained by a low number of cells; cells taken from degenerated discs are often senescent and dysfunctional, so they cannot be used for therapy [120]. Allogeneic or xenogeneic cell sources appear promising in preclinical studies but still require rigorous clinical and ethical validation [121,122]. At present, the most practical way is to use mesenchymal stem cells (MSCs) from easily obtainable sources such as bone marrow, fat tissue, etc., which have the advantage of being easy to obtain and manage ethically [16]. Although extensive preclinical studies suggest that stem cell injection can enhance ECM synthesis and modulate the immune microenvironment, multiple issues must be resolved before routine clinical adoption. Importantly, most clinical evidence remains based on small samples, early-phase trials, or case series [105,106,107,109]. While these reports suggest favorable safety and symptom relief, efficacy and long-term outcomes require confirmation in larger, standardized randomized controlled trials (RCTs). Key barriers include the harsh disc microenvironment (avascularity, hypoxia, acidity, and nutrient limitation), which can compromise transplanted cell survival and function [13,16,17]. In addition, MSC heterogeneity across tissue sources (bone marrow, adipose, synovium) and across preparation/expansion protocols leads to variable secretomes and differentiation capacity, complicating outcome predictability [4,104,120]. Finally, inconsistencies in study design, outcome measures, and follow-up duration further highlight the need for larger, harmonized RCTs to establish both efficacy and long-term safety [105,106,107,109] (Table 3).

### 6.2. Application of Transgenic Technology in IVDR

Gene therapy strategies introduce therapeutic genes into disc cells via delivery vectors and have been actively explored as an IVDR strategy. As an in vivo proof-of-concept, Liang et al. demonstrated that intradiscal injection of an adenoviral vector in a mouse disc degeneration model led to localized transgene expression [123]. Regarding therapeutic efficacy, Luo et al. delivered the GDF-5 gene into human nucleus pulposus cells in vitro using an adenovirus, which upregulated ECM components such as aggrecan and type II collagen [124]. To improve safety, Lattermann et al. tested adeno-associated virus (AAV) vectors in rabbits and showed that AAV can achieve intradiscal transgene expression with lower immunogenicity than first-generation adenoviral vectors [125]. Overall, these studies support the feasibility of both viral and non-viral gene delivery systems for enhancing ECM synthesis and promoting disc repair.

Viral vectors are widely used in IDD gene therapy because of their high transduction efficiency and robust transgene expression. Commonly used systems include adenovirus, AAV, and retrovirus. Early transgenic studies frequently employed adenoviral vectors. For example, Liang et al. reported that adenoviral injection into mouse lumbar discs enabled gene transfer relevant to lumbar disc degeneration treatment [123]. Luo et al. found that adenovirus-mediated delivery of GDF-5 promoted ECM production in degenerative human nucleus pulposus cells [124]. In mouse models, adenoviral vectors carrying GDF-5 increased nucleus pulposus/annulus fibrosus cell proliferation and ECM synthesis [126]. Wallach et al. engineered an adenoviral vector carrying TIMP and observed increased proteoglycan content in degenerated human disc cells after in vitro transfection [127]. Yoon et al. further reported that adenoviral transfer of LMP-1 increased proteoglycan content in disc matrix in vitro and in vivo [128]. Additional work suggests that transcriptional regulators (e.g., SOX9) may be delivered to promote collagen and cartilage-like matrix production in degenerated nucleus pulposus cells [89].

Although adenoviral vectors can elicit host immune responses, AAV has become a common choice in IDD research due to relatively low immunogenicity and toxicity [129]. Ren et al. reported increased proteoglycan expression after AAV-mediated co-transduction of BMP-1 and SOX9 in a rabbit model of disc degeneration [130]. Lattermann et al. also showed that AAV vectors can efficiently transfect disc cells with robust transgene expression [125]. Serotype selection may matter: AAV6 has been suggested as a promising option because it shows favorable properties in nucleus pulposus cells [131]. For instance, AAV6-mediated ADAMTS-4 knockdown increased aggrecan expression without inducing obvious toxic or inflammatory catabolic responses in degenerative human nucleus pulposus cells [132].

Non-viral vectors (e.g., liposomes and cationic polymers) offer potential advantages in reducing immunogenicity, toxicity, and insertional mutagenesis risk, though transfection efficiency is generally lower [87]. Chung et al. reported that liposome-mediated telomerase gene delivery in sheep nucleus pulposus cells could delay senescence and increase ECM production [133]. Wang et al. found that liposomal transfection of circRNA SEMA4B increased proteoglycan production and helped mitigate nucleus pulposus degeneration [134]. Morrey et al. screened multiple non-viral carriers and identified liposome–protein–polyamine complexes as promising systems for disc gene delivery [135]. As an example of an injectable delivery platform, AQP3-lipo@GelMA has been developed for intradiscal administration, providing sustained release and promoting repair after mechanical compression injury [90] (Table 4).

### 6.3. Application of Biomaterials in IVDR

IDD was initially considered an age-related disorder; however, it is now understood to be driven by intracellular processes, extracellular microenvironmental changes, mechanical loading, and genetic factors [139]. The extracellular microenvironment provides both physical and chemical cues that shape cell fate, survival, and matrix production. In healthy discs, the ECM is rich in water, proteoglycans, elastin, glycoproteins, and glycosaminoglycans [140,141]. Therefore, biomaterials that mimic ECM composition and mechanics (e.g., hydrogels and polysaccharide-based materials) represent potential therapeutic strategies for IDD. Current candidates include hydrogels, nanomaterials, polyphenolic organic materials, and inorganic antioxidant materials [142]. Hydrogels and nanomaterials have shown promise in preclinical studies for cell delivery and/or controlled release of anti-inflammatory agents [143,144]. For example, antioxidant polyphenols such as curcumin have been reported to reduce pain-related behavior in rat models [145,146].

Hydrogels are among the most advanced biomaterials designed to resemble the nucleus pulposus microenvironment, featuring high water content, good biocompatibility, and tunable mechanical properties (e.g., stiffness and swelling) [143]. These properties support their use as nucleus pulposus substitutes or as platforms for controlled delivery of cells and bioactive molecules. Ligorio et al. developed a graphene oxide/self-assembling peptide hybrid hydrogel loaded with TGF-β3, which supported nucleus pulposus–like differentiation of encapsulated MSCs and inhibited pro-inflammatory NF-κB signaling in vitro [144]. Three-dimensional (3D) bioprinting of hydrogels further enables precise deposition of cell-laden constructs and may facilitate fabrication of complex disc-like structures [147]. Polysaccharide-based materials (e.g., hyaluronic acid, alginate, chitosan, chondroitin sulfate) are also promising due to biocompatibility, biodegradability, and their ability to mimic natural ECM [148]. Given that degenerative discs are often acidic due to lactate accumulation from long-term glycolysis, pH-responsive hydrogels that release therapeutics in response to local acidity may offer added benefits [149]. Hydrogels can also serve as delivery vehicles for anti-inflammatory factors (e.g., IL-1 receptor antagonist) and anabolic factors (e.g., TGF-β) to inhibit NF-κB signaling and rebalance cytokine networks [144].

Nanomaterials offer a large surface area and tunable surface chemistry, enabling targeted delivery and improved penetration of biological barriers, including strategies aimed at oxidative stress within nucleus pulposus cells [142]. Because mitochondrial dysfunction contributes to ECM degradation and nucleus pulposus cell loss, mitochondria-protective nanomedicines have become an emerging direction for disc repair [150,151]. Nucleus pulposus-targeted nanocarriers may support more precise intradiscal drug delivery and delay degeneration. For example, miRNA therapeutics packaged in targeted nanoparticles have been proposed to modulate inflammatory signaling pathways (e.g., JAK1/STAT3 and IL-6/IL-1β/TNF axes) and anabolic regulators (e.g., SIRT1), thereby alleviating IDD [88,91] Membrane-coated nanoparticles may further improve targeting by preserving receptor–ligand interactions relevant to inflammatory mediators, offering a potential strategy to modulate senescence-associated secretory phenotypes. In addition, glutathione-doped carbon dots (GSH-CDs), as antioxidant nanoenzymes, have been reported to reduce oxidative stress, limit ROS-driven senescence, and alleviate IDD progression [152].

Natural polyphenolic compounds have been investigated for disc repair because of their antioxidant and anti-inflammatory activities. In a rat lumbar disc degeneration model, Ma et al. reported that intragastric curcumin reduced pain-related behavior and histological degeneration, mechanistically linked to inhibition of NF-κB activation and inflammatory cytokine production [146]. At the cellular level, resveratrol has been reported to protect nucleus pulposus cells from apoptosis by scavenging intracellular ROS [153]. Together, these studies suggest that polyphenols (e.g., curcumin and resveratrol) can mitigate oxidative stress and inflammation, which are key drivers of IDD [142]. Resveratrol, a polyphenol enriched in grape skins, shows anti-inflammatory and antioxidative effects and may reduce ROS-induced mitochondrial dysfunction and apoptosis in nucleus pulposus cells [151,153,154]. Curcumin (from turmeric) has been reported to slow IDD progression in rats by inhibiting NF-κB-p65 translocation and reducing inflammatory cytokine secretion [145,146]. Polyphenol-derived functional materials (e.g., EGCG-based metallopolyphenol systems) can integrate antioxidant, anti-apoptotic, anti-inflammatory, and ROS-scavenging properties [155]. EGCG has also been reported to protect disc cells from oxidative stress and may alleviate IDD-associated pain [156].

**Figure 2:**
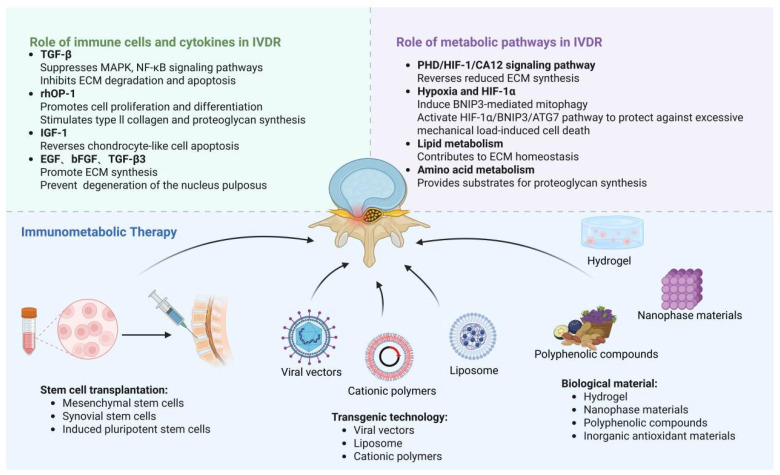
Therapeutic landscape of IVDR via immunometabolic modulation. Achieving IVDR requires harnessing the body’s endogenous repair capacity by manipulating key immunometabolic pathways. Pro-regenerative cytokines and growth factors (e.g., TGF-β, rhOP-1, IGF-1) promote anabolism by supporting cell proliferation and extracellular matrix (ECM) synthesis. Meanwhile, metabolic pathways such as HIF-1α signaling and mitophagy play a key role in maintaining cell survival and matrix homeostasis within the disc’s harsh microenvironment. These basic mechanisms provide a rationale for emerging therapeutic strategies, including stem cell transplantation (MSCs, iPSCs), gene-based approaches (viral and non-viral vectors), and advanced biomaterials (hydrogels, nanomaterials, polyphenols, etc.). By synergistically targeting these pathways, these approaches aim to restore the structure and function of the degenerated intervertebral disc.

Inorganic antioxidant nanomaterials (e.g., cerium oxide and selenium nanoparticles) exhibit high chemical stability and can mimic endogenous antioxidant enzymes such as catalase and superoxide dismutase, enabling prolonged anti-oxidative activity [157,158]. Cerium oxide nanozymes have been reported to reduce intracellular ROS, slow cellular senescence, decrease apoptosis, and support nucleus pulposus cell metabolism with relatively low cytotoxicity [159]. In IL-1β–induced inflammatory conditions, selenium nanoparticles have been reported to preserve nucleus pulposus matrix synthesis (higher aggrecan and type II collagen) while reducing catabolic enzymes (MMP-13 and ADAMTS5) and supporting mitochondrial redox/energy homeostasis [158] (Figure 2) (Table 5).

### 6.4. Translation Barriers and Limitations of Current Immunometabolic Therapies

Although immunometabolic therapies show promise, multiple barriers still impede translation from bench to bedside. A clear understanding of these limitations is essential for designing effective translational pathways.

First, much of the current evidence is derived from animal models (e.g., rodent needle puncture or compression models) or in vitro cell experiments [23,94,123]. While indispensable, these systems often fail to reproduce the decades-long, multifactorial trajectory of human IDD and its unique biomechanical/biochemical microenvironment. For example, severe neovascularization and inflammatory cell infiltration observed in advanced human disc degeneration may not be simultaneously recapitulated in common animal models [18,41,126]. Therefore, more clinically relevant models (e.g., aged animals, organ culture under dynamic loading, human disc organoids) are needed to improve predictive validity [17,60,139].

Second, therapy standardization remains a major challenge, especially for cell therapies. MSC properties vary substantially with tissue source (bone marrow, adipose, synovium), donor status, and culture/expansion protocols, resulting in heterogeneous secretomes and differentiation capacity and thus variable therapeutic outcomes [4,96,104,120]. Similarly, biomaterial properties (e.g., degradation rate, mechanical behavior, growth factor release kinetics) can vary between batches and studies, making it difficult to compare results and to define standardized dosing [142,144,148]. Establishing rigorous characterization criteria and standardized manufacturing/quality-control workflows is therefore critical for reproducibility and translation.

Third, the pathological disc microenvironment itself is a therapeutic barrier. The target tissue is avascular, hypoxic, acidic, nutritionally limited, and mechanically loaded—conditions that jeopardize transplanted cell survival and the stability/retention of delivered biomolecules [13,16,17]. Thus, simple injection may lead to rapid cell apoptosis or drug clearance. Intelligent delivery systems (e.g., pH-responsive hydrogels, protective scaffolds that enhance retention and support cell viability) are increasingly essential rather than optional [119,143,144,149].

Finally, early clinical outcomes remain inconsistent, ranging from substantial to modest effects [105,106,107]. This variability reflects the multifactorial nature of chronic low back pain, which may involve genetic influences (e.g., pain-related polymorphisms), psychosocial factors, central sensitization, and coexisting spinal pathologies beyond disc degeneration alone [21,73,91]. Future clinical trials should incorporate biomarker-based and phenotype-based patient stratification, together with long-term follow-up, to clarify responders/non-responders and to monitor risks such as abnormal tissue hyperplasia or immune reactions to allogeneic products [107,121,122].

## 7. Conclusions and Future Prospects

IDD can be viewed as an immunometabolic disease, in which immune dysregulation and metabolic dysfunction reinforce each other, forming a vicious cycle that drives disc degeneration and clinical symptoms. Under this conceptual shift, stem cell transplantation, gene therapy, biomaterial implants, and other high-tech therapies should be regarded as active reprogrammers of the degenerated disc microenvironment, not passive add-ons. Their therapeutic potential lies in tipping the local balance from catabolism and inflammation toward anabolism and tissue repair.

Future progress will require overcoming several key barriers. A critical next step is to translate promising preclinical strategies into well-designed early-phase clinical trials with biomarker-driven readouts and patient stratification, thereby providing a robust proof of concept while rigorously assessing safety. First, the field should shift from broad immunosuppression to immunomodulation that promotes a reparative immune phenotype. Second, engineered therapies must survive and function within the harsh avascular, hypoxic, and acidic microenvironment of the disc. Finally, integrated multi-omics approaches will be needed to develop personalized therapeutic strategies for individual patients based on their immunometabolic signatures.

Ultimately, by targeting the core immunometabolic drivers of IDD, it may become feasible to pursue biological disc restoration beyond the more modest aim of symptom control alone. This shift in perspective may also inform therapeutic development, not only for spinal disorders but also for other chronic degenerative diseases that share similar immunometabolic mechanisms.

## Figures and Tables

**Table 1:** Role of immune cells and cytokines in intervertebral disc degeneration.

Reference	Immune Cells/Inflammatory Mediators	Key Findings
Shamji et al. (2010) [40]	Macrophages, lymphocytes	Degenerative disc tissue is characterized by the infiltration of macrophages and lymphocytes
Miyamoto et al. (2000) [42]	TNF, ILs, MMPs	Pro-inflammatory cytokines are upregulated in degenerated discs, resulting in reduced proteoglycan content and accelerated ECM degradation
Li et al. (2016) [43]	TNF, ILs, MMPs	Inflammatory mediators cause a drop in PG levels in the intervertebral disc
Wang et al. (2020) [34]	IL-1α, IL-1β	IL-1α and IL-1β activate NF-κB/MAPK signaling, upregulating MMPs and ADAMTS and promoting ECM degradation
Dowdell et al. (2017) [44]	MMP-1, MMP-3, MMP-13, ADAMTS-4	These degradative enzymes are expressed significantly more in degenerated discs and play an important role in the degeneration of the disc
Johnson et al. (2015) [37]	IL-1β	Elevated IL-1β promotes MMP production, inhibiting proteoglycan and collagen synthesis and leading to ECM degradation
Le Maitre et al. (2005) [45]	IL-1β	This study provided the first evidence that IL-1 is a major mediator in human disc degeneration, showing that it strongly induces catabolic enzymes (MMPs, ADAMTS, COX-2) and inhibits matrix synthesis.
Cherif et al. (2024) [46]	IL-1	Higher IL-1 levels modulate the expression of degradative enzymes and change ECM composition in disc degeneration
Zhang et al. (2022) [53]	TNF	TNF can cause inflammatory cytokines to be produced, ECm to be degraded, and cells to die through the NF-κB/MAPK pathways
Yao et al. (2016) [39]	IL-17	IL-17 induces MMP-13 and type I collagen but decreases the synthesis of type II collagen, leading to a disruption in the ECM, which exacerbates IDD
Xiang et al. (2024) [19] Wang et al. (2016) [47]	IL-1β, IL-6, TNF	These SASP components establish a local inflammatory microenvironment that promotes disc cell senescence and immune cell infiltration
Rannou et al. (2000) [48]	IL-1β	IL-1β promotes MMP mRNA production, PGE2 production, and ECM degradation in annulus fibrosus cells.
Das et al. (2019) [49]	PGE2	As a key inflammatory marker, PGE2 can induce sciatica and enhance MMP-3 activity, thereby accelerating ECM degradation
Teixeira et al. (2023) [50] Hu et al. (2023) [51]	IL-8	Elevated IL-8 levels in degenerative discs are correlated with IDD development
Shi et al. (2019) [11]	Macrophages	Macrophages can undergo phenotypic switching from a pro-inflammatory (M1) to a tissue-repair (M2) phenotype in response to acute injury
Nakazawa et al. (2018) [41]	Macrophages	The presence of M1 and M2c macrophages correlates with the severity of degeneration, implying their roles in both inflammation and repair
Yang et al. (2019) [52]	Macrophages (M1)	M1 macrophages and disc cells form a synergistic interaction that intensifies local inflammation and promotes the degenerative process

**Note**: TNF: tumor necrosis factor; IL: interleukin (ILs: interleukins); MMPs: matrix metalloproteinases; PGs: proteoglycans; PGE2: prostaglandin E2; ECM: extracellular matrix; NF-κB/MAPK pathway: nuclear factor-κB/mitogen-activated protein kinase pathway; ADAMTS: a disintegrin and metalloproteinase with thrombospondin motifs; SASP: senescence-associated secretory phenotype; IDD: intervertebral disc degeneration.

**Table 2:** Role of metabolic pathways in intervertebral disc degeneration.

Reference	Metabolic Pathways/Factors	Key Findings
Fan et al. (2023) [54] Song C et al. (2024) [55] Song Y et al. (2021) [56] Urban et al. (2004) [17]	Glycolysis, lactate	As a result of the avascular and hypoxic disc microenvironment, nucleus pulposus cells are forced to use glycolysis which leads to lactate accumulation and redox imbalance
Feng et al. (2017) [35]	ROS, mitochondrial dysfunction	Mitochondrial dysfunction is a primary source of excessive ROS generation and promotes the progression of IDD via the induction of inflammation and impairing autophagy
Akanji et al. (2021) [57]	Redox Homeostasis	Disruption of redox homeostasis is an important pathogenic mechanism leading to the development and propagation of IDD
Nishimura et al. (2021) [58]	Oxidative Stress	Oxidative stress, driven by an imbalance between ROS production and antioxidant capacity, disrupts cellular metabolism and impairs cell function; it may also contribute to pain via effects on the nervous system
Hollander et al. (2020) [59]	ROS	Excessive ROS accumulation induces oxidative stress, leading to macromolecular damage, loss of cellular integrity, and metabolic dysregulation
Kodama et al. (2023) [60]	Nutrient Supply, Glycolysis	Under limited nutrient availability, reducing the metabolic demand of NP cells or improving glycolytic efficiency may decrease cell death and mitigate IDD progression
Kim et al. (2021) [13]	Hypoxia, Glycolysis, Acidity	The acidic microenvironment resulting from anaerobic metabolism suppresses glycolysis and oxygen utilization, accelerating ECM degradation and cell death
Yi et al. (2023) [6]	Lipid Metabolism, Adipokines	Disrupted lipid metabolism contributes to IDD pathogenesis by modulating inflammation, cellular catabolism, tissue calcification, and apoptosis
Liu et al. (2015) [61]	Lipid Metabolism Disorders	These disorders are characterized by dysregulated adipokine secretion and accumulation of deleterious fatty acids and cholesterol
Curic et al. (2020) [36]	Adipokines, Fatty Acids, Cholesterol	These lipid metabolites promote IDD by reshaping the inflammatory milieu, enhancing cellular catabolism and calcification, and inducing apoptosis
Jin et al. (2020) [62]	Estrogen	Reduced estrogen levels promote IDD by inducing oxidative and ER stress, thereby enhancing ECM degradation and apoptosis while decreasing type II collagen
Sobajima et al. [63]	Amino Acid Metabolism, osteopontin-1	Disrupted amino acid metabolism (e.g., downregulated osteopontin-1) leads to an imbalance in protein synthesis and degradation, exacerbating ECM destruction and reducing cell viability.

**Note**: ROS: reactive oxygen species; ECM: extracellular matrix; ER: endoplasmic reticulum; IDD: intervertebral disc degeneration.

**Table 3:** Applications of stem cell transplantation in intervertebral disc regeneration.

Reference	Therapies/Mechanisms	Key Findings
Wang et al. (2014) [92] Jin et al. (2013) [93]	Stem cell transplantation	Stem cell transplantation has been shown to restore disc structure and improve intervertebral disc function, thereby supporting IVDR
Feng et al. (2011) [94] Serigano et al. (2010) [95] Anderson et al. (2013) [96] Chun et al. (2012) [97] Allon et al. (2010) [98]	Stem cell therapy	Preclinical animal studies confirm that stem cell therapy promotes IVDR by restoring disc height, stimulating ECM synthesis, and improving disc function
Bhujel et al. (2022) [4]	MSCs	MSCs can differentiate toward NP-like cells while exerting immunomodulatory and anti-catabolic effects
Isgro et al. (2014) [101] Miller et al. (2012) [102]	Conservative management	Represents first-line management for early-stage IDD, primarily focusing on symptom control
Wang et al. (2014) [103]	Surgical intervention	Fails to reverse or delay the degenerative process, as it does not address the underlying pathology
Ohnishi et al. (2023) [104]	MSCs and biological scaffolds	Represents a WHO-endorsed regenerative approach, with clinical trials primarily focusing on MSCs and biological scaffolds
Hingert et al. (2020) [111]	MMPs and TIMPs	The balance between MMPs and TIMPs is crucial for disc tissue remodeling; upregulation of TIMPs may help restore the catabolic/anabolic balance
Le et al. (2004) [112]	TIMPs	TIMPs have been identified as potential therapeutic targets for promoting IVDR
Zhu et al. (2020) [113]	sEVs	sEVs derived from iPSC-MSCs modulate the disc microenvironment by downregulating catabolic markers (ADAMTS-4, MMP-3) and upregulating anabolic markers (proteoglycans and collagen)
Yoshikawa et al. (2010) [105]	Autologous bone marrow MSCs	Autologous bone marrow–derived MSC transplantation promotes disc tissue regeneration, demonstrating significant therapeutic benefits for degenerative disc disease
Watanabe et al. (2010) [108]	MSCs co-culture	Direct co-culture with MSCs significantly enhances the proliferative capacity of human nucleus pulposus cells
Meisel et al. (2006) [109] Meisel et al. (2007) [106]	Stem cell transplantation (clinical studies)	Clinically shown to markedly alleviate low back pain, preserve disc height, and increase water content in the disc/adjacent discs
Coric et al. (2013) [107]	Stem cell transplantation (prospective trial)	A prospective clinical trial confirmed its efficacy in alleviating low back pain and promoting functional restoration of the disc, with a favorable safety profile
Miyamoto et al. (2010) [100]	Human MSCs co-culture	Modulates NP-cell gene expression, reducing degradative enzymes and inflammatory factors while promoting ECM synthesis.
Crevensten et al. (2004) [110]	Allogeneic MSCs transplantation	In a rat model, transplanted MSCs exhibited significant proliferation, leading to increased disc height and enhanced proteoglycan production, indicating a reparative effect.
Korecki et al. (2010) [114]	MSCs co-culture	Co-culture with MSCs significantly increases proteoglycan synthesis in chondrocytes, highlighting their anabolic potential for disc regeneration
Steck et al. (2005) [99]	MSCs in TGF-β medium	Differentiated cells exhibited a nucleus pulposus-like phenotype and secreted type II collagen and proteoglycans
Chen et al. (2009) [115]	Synovial stem cells with TGF-β	Higher concentrations of TGF-β induced differentiation toward an NP-like phenotype, characterized by markedly increased proteoglycan synthesis
Le et al. (2006) [116]	MSCs co-culture with annulus fibrosus/nucleus pulposus cells	Stimulated robust proliferation in both annulus fibrosus and nucleus pulposus cells; however, proteoglycan production increased only in annulus fibrosus cells co-cultured with MSCs
Harfe et al. (2022) [117]	iPSCs	iPSCs can be differentiated into NP-like cells, offering a promising cell source for IVDR
Tan et al. (2024) [118]	MSC-derived exosomes	Nanoscale extracellular vesicles that mediate intercellular communication
Luo et al. (2022) [119]	Exosomes	Ameliorate IDD through antioxidant, anti-inflammatory, and anti-endoplasmic reticulum (ER) stress effects in nucleus pulposus cells
Bhujel et al. (2022) [4]	Exosomal miR-223	Regulates nucleus pulposus cell fate by inhibiting proliferation, upregulating anabolic genes (aggrecan, collagen II), and downregulating catabolic enzymes and NF-κB signaling
Vasiliadis et al. (2014) [120]	Autologous disc stem cells	Source acquisition is a major challenge: extraction from healthy discs causes iatrogenic damage, while cells from degenerative discs exhibit premature senescence and poor therapeutic potential
Nomura et al. (2001) [121] Sato et al. (2003) [122]	Allogeneic/xenogeneic stem cells	Show promising outcomes in animal studies, but clinical trial evidence remains limited due to ethical and safety concerns
Hickman et al. (2022) [16]	MSCs	Offer diverse and accessible sources (e.g., bone marrow, adipose tissue), partially circumventing ethical dilemmas and facilitating clinical application.

**Note**: IVDR: intervertebral disc regeneration; ECM: extracellular matrix; MSCs: mesenchymal stem cells; IDD: intervertebral disc degeneration; NF-κB: nuclear factor-κB; ADAMTS: a disintegrin and metalloproteinase with thrombospondin motifs; WHO: World Health Organization; TIMPs: tissue inhibitors of metalloproteinases; sEVs: small extracellular vesicles; iPSCs: induced pluripotent stem cells; MMPs: matrix metalloproteinases; TGF: transforming growth factor; NP: nucleus pulposus.

**Table 4:** Applications of transgenic technology in intervertebral disc regeneration.

Reference	Vectors/Genes	Key Findings
Liang et al. (2010) [123]	Adenovirus	Demonstrated that adenoviral vectors are promising gene delivery vehicles for treating lumbar disc degeneration in a mouse model
Luo et al. (2016) [124]	Adenovirus (GDF-5)	Adenovirus-mediated GDF-5 gene transfer promoted ECM synthesis in human NP cells
Cui et al. (2008) [126]	Adenovirus (GDF-5)	In a mouse model, GDF-5 gene therapy enhanced the proliferation of NP and AF cells and increased ECM synthesis
Wallach et al. (2003) [127]	Adenovirus (TIMP)	Transfection with a TIMP- expressing adenovirus led to increased proteoglycan synthesis in human degenerated disc cells
Yoon et al. (2004) [128]	Adenovirus (BMP-1)	Adenovirus-mediated BMP-1 gene transfer significantly increased matrix proteoglycan content in rabbit intervertebral discs
Wang et al. (2011) [136]	BMP-7	In chondrocytes, BMP-7 stimulated both proteoglycan and type II collagen synthesis
Paul et al. (2003) [89]	Adenovirus (SOX9)	Adenoviral delivery of the SOX9 gene increased collagen synthesis in degenerated human NP cells
Gonçalves et al. (2004) [129]	AAV	AAV is widely used in IDD research due to its low immunogenicity and non-pathogenic nature
Ren et al. (2013) [130]	AAV (BMP-1 + SOX9)	AAV-mediated co-transduction of BMP-1 and SOX9 enhanced proteoglycan expression in a rabbit IDD model
Lattermann et al. (2005) [125]	AAV	AAV vectors achieve efficient transfection and robust transgene expression in lumbar disc cells, representing a potentially safer viral vector
Mern et al. (2015) [131]	AAV6	AAV6 is a preferred serotype for IDD gene therapy, as it does not exacerbate inflammation or catabolism
Mern et al. (2017) [132]	AAV6 (ADAMTS4 knockdown)	AAV6-mediated ADAMTS4 silencing induced a long-term increase in aggrecan levels without detectable toxic or inflammatory responses
Wehling et al. (1997) [137]	Retrovirus (LacZ, IL-1RA)	Demonstrated the feasibility of exogenous gene transfer into adult animal intervertebral discs using retroviral vectors
Priyadarshani et al. (2016) [87]	Non-viral vectors	Provide a safer alternative to viral vectors by reducing immunogenicity, toxicity, and insertional mutagenesis risk, despite lower transfection efficiency
Chung et al. (2007) [133]	Liposomes (Telomerase)	Liposomal delivery of the telomerase gene delayed cellular senescence and promoted ECM production in sheep NP cells.
Wang et al. (2018) [134]	Liposomes (circRNA SEMA4B)	Liposomal transfection of circRNA SEMA4B increased proteoglycan production, helping to prevent NP degeneration
Morrey et al. (2008) [135]	Liposome complexes	Identified liposome-protein-polyamine complexes as a promising non-viral vector platform for IDD gene therapy
Cui et al. (2024) [138]	AQP3 inhibition	AQP3 inhibition reduces mitochondrial respiratory activity, leading to mitochondrial dysfunction and promoting cellular senescence and apoptosis
Hu et al. (2025) [90]	AQP3-lipo@GelMA	An injectable hydrogel system designed to enhance NP cell self-renewal capacity, thereby promoting regeneration of degenerated discs

**Note**: GDF: growth differentiation factor; circRNA SEMA4B: circular RNA semaphorin 4B; TIMP: tissue inhibitors of metalloproteinases; ECM: extracellular matrix; IL-1RA: interleukin-1 receptor antagonist; AAV: adeno-associated virus; BMP: bone morphogenetic protein; IDD: intervertebral disc degeneration; SOX: SRY-related high-mobility group box; AQP: aquaporin; GelMA: methacrylated gelatin; ADAMTS: a disintegrin and metalloproteinase with thrombospondin motifs; NP: nucleus pulposus; AF: annulus fibrosus.

**Table 5:** Applications of biomaterials in intervertebral disc regeneration.

Reference	Biomaterials/Categories	Key Findings
Liu et al. (2026) [139]	Etiology of IDD	IDD is recognized as a multifactorial disease influenced not only by aging, but also by cell-intrinsic mechanisms, the extracellular microenvironment, and genetic factors
Zhang et al. (2022) [140] Roughley et al. (2004) [141]	ECM	The ECM, composed primarily of water, proteoglycans, and collagens, provides a structural scaffold and biochemical cues that regulate cellular activity
Mai et al. (2025) [142]	Hydrogels, nanomaterials, polyphenols, inorganic	These antioxidant biomaterials are widely investigated in IDD for ECM mimicry and controlled delivery of emerging therapeutics
Desai et al. (2024) [143]	Hydrogels	Hydrogels exhibit biomimetic properties similar to the human NP, including biocompatibility, tunable mechanics, and high water retention
Liu et al. (2023) [160]	Hydrogels	Their high water content provides a favorable aqueous microenvironment for intervertebral disc cell growth and function
Zhang et al. (2024) [147]	3D-printed hydrogels	Enable precise, layer-by-layer deposition of cell-laden biomaterials, offering a novel approach for constructing disc-mimetic tissues
Wang et al. (2025) [148]	Polysaccharide-based materials	Promising candidates for IVDR due to their biocompatibility, biodegradability, and ability to mimic the native NP-ECM
Wang et al. (2024) [149]	Responsive hydrogels	Stimuli-responsive hydrogel systems that respond to the acidic microenvironment of degenerated discs offer significant potential for on-demand, targeted drug release
Ligorio et al. (2021) [144]	Hydrogels as anti-inflammatory agents	Can serve as delivery vehicles for anti-inflammatory agents (e.g., IL-1RA, TGF-β) to inhibit NF-κB signaling and modulate cytokine networks
Mai et al. (2025) [142]	Nanomaterials	Their high surface-area-to-volume ratio and ability to penetrate biological barriers allow targeted modulation of oxidative stress in NP cells
Shi et al. (2024) [150] Lai et al. (2019) [151]	Nanoparticles	Represent a promising therapeutic strategy for protecting and restoring mitochondrial function, a central driver in IDD pathogenesis
Liu et al. (2024) [88] Risbud et al. (2014) [91]	NP-targeted nanocarriers	Enable precise delivery of therapeutics (e.g., miRNAs, cytokine adsorbers) to preserve disc function by modulating key signaling pathways and cellular senescence
Bu et al. (2024) [152]	GSH-CD	A novel antioxidant nanozyme that ameliorates oxidative stress and inhibits NP cell senescence by mimicking multiple antioxidant enzyme activities
Mai et al. (2025) [142]	Polyphenolic compounds (e.g., curcumin, resveratrol)	Natural antioxidants that protect NP cells from oxidative stress by scavenging free radicals and reducing inflammation
Lai et al. (2019) [151]	Polyphenols	Their intrinsic antioxidant properties make them promising therapeutic candidates for treating IDD
Liu et al. (2022) [154]	Resveratrol	A grape-derived polyphenol with both anti-inflammatory and antioxidant effects
Li et al. (2018) [153]	Resveratrol	Effectively reduces intracellular ROS levels in NP cells and inhibits apoptosis
Xiao et al. (2017) [145]	Curcumin	A natural pigment with a favorable safety profile, exhibiting anti-inflammatory and antioxidant properties
Ma et al. (2015) [146]	Curcumin	In a rat model, curcumin slows IDD progression by inhibiting NF-κB signaling, thereby reducing inflammatory cytokine release
Zhou et al. (2024) [155]	Polyphenol-metal ion polymers	Coordination of EGCG with copper ions yields multifunctional materials with antioxidant, anti-apoptotic, and anti-inflammatory capabilities
Krupkova et al. (2016) [156]	EGCG	Alleviates IDD-induced pain and counteracts oxidative stress by protecting mitochondria from membrane depolarization
Behroozi et al. (2024) [157] He et al. (2024) [158]	Inorganic materials	Exhibit chemical stability and can mimic natural enzymes (e.g., catalase, SOD) to provide sustained antioxidant effects
Chang et al. (2025) [159]	Cerium oxide nanoenzymes	Effectively mitigate IDD by reducing intracellular ROS, delaying senescence, decreasing apoptosis, and improving NP cell metabolism with low cytotoxicity
He et al. (2024) [158]	Selenium nanoparticles	Protect NP cells under inflammatory conditions by preserving matrix synthesis and maintaining mitochondrial structural and metabolic integrity via potent antioxidant activity

**Note**: IDD: intervertebral disc degeneration; ECM: extracellular matrix; 3D: three-dimensional; IVDR: intervertebral disc regeneration; IL-1RA: interleukin-1 receptor antagonist; TGF: transforming growth factor; NF-κB: nuclear factor-κB; GSH-CD: glutathione-doped carbon dots; ROS: reactive oxygen species; EGCG: epigallocatechin gallate; MMP: matrix metalloproteinase; ADAMTS: a disintegrin and metalloproteinase with thrombospondin motifs; SOD: superoxide dismutase; NP: nucleus pulposus.

## Data Availability

No new data were created or analyzed in this study. Data sharing is not applicable to this article.
